# The 2025 European Society of Cardiology guidelines for myocarditis and pericarditis and the evolving role of cardiovascular magnetic resonance

**DOI:** 10.1016/j.jocmr.2025.102674

**Published:** 2025-12-31

**Authors:** Jeanette Schulz-Menger, Jan Gröschel, Vanessa M. Ferreira, Jan Bogaert, Chiara Bucciarelli-Ducci, Massimo Imazio, Matthias G. Friedrich

**Affiliations:** Charite-Universitätsmedizin Berlin, Freie Universität Berlin and Humboldt Universität zu Berlin, Charitéplatz 1, 10117 Berlin, Germany; Working Group on Cardiovascular Magnetic Resonance, Experimental and Clinical Research Center, a Joint Cooperation Between Charité Medical Faculty and the Max-Delbrück Center for Molecular Medicine, Berlin, Germany; DZHK (German Centre for Cardiovascular Research), Partner Site Berlin, Berlin, Germany; Department of Cardiology and Nephrology, HELIOS Klinikum Berlin-Buch, Berlin, Germany; Charite-Universitätsmedizin Berlin, Freie Universität Berlin and Humboldt Universität zu Berlin, Charitéplatz 1, 10117 Berlin, Germany; Working Group on Cardiovascular Magnetic Resonance, Experimental and Clinical Research Center, a Joint Cooperation Between Charité Medical Faculty and the Max-Delbrück Center for Molecular Medicine, Berlin, Germany; DZHK (German Centre for Cardiovascular Research), Partner Site Berlin, Berlin, Germany; Deutsches Herzzentrum der Charité - Department of Cardiology, Angiology and Intensive Care Medicine, Charitéplatz 1, 10117 Berlin, Germany; University of Oxford Centre for Clinical Magnetic Resonance Research, Division of Cardiovascular Medicine, Radcliffe Department of Medicine, University of Oxford, Oxford, United Kingdom; Department of Radiology, University Hospitals Leuven, Leuven Belgium; Department of Imaging and Pathology, KU Leuven, Leuven, Belgium; Royal Brompton and Harefield Hospitals, Guys’ & St Thomas NHS Trust, London, UK; KingdomSchool of Biomedical Engineering and Imaging Sciences, Faculty of Life Sciences and Medicine, King’s College University, London, UK; National Heart and Lung Institute, Imperial College London, London, UK; Department of Medicine, University of Udine, Udine, Italy; Cardiothoracic Department, University Hospital Santa Maria della Misericordia, Udine, Italy; Departments of Medicine and Diagnostic Radiology, McGill University Health Centre, Montreal, Quebec, Canada

## 1 Introduction

The 2025 European Society of Cardiology (ESC) Guidelines on Inflammatory Myocardial and Pericardial Syndromes (IMPS) provide, for the first time, a unified framework that integrates myocarditis, pericarditis, and overlapping entities such as myopericarditis and perimyocarditis under one conceptual umbrella [1]. IMPS represent a heterogeneous group of conditions that have long challenged both clinicians and researchers. This editorial highlights the key messages from the new guidelines, with particular emphasis on the transformative role of non-invasive imaging, especially cardiovascular magnetic resonance (CMR), in reshaping our diagnostic and management strategies. The proposed paradigm shift, with CMR at the forefront, with a Class IB indication for assessing suspected IMPS, highlights the possibility of providing a definite diagnosis of “CMR-proven” myocarditis in most cases. The following sections summarize the key messages and provide details on the role of CMR in IMPS. Importantly, ESC guidelines were developed using a standardized, scientifically rigorous process, including stringent proof of all available peer-reviewed evidence as assessed by expert methodologists, with three review rounds with nearly 10,000 comments from at least 40 and up to 100 reviewers, and a blinded voting for the recommendation. The exact methodology is available in the ESC Guidelines document [2].

## 2 Summary of key messages

### 2.1 IMPS: an umbrella concept

By explicitly recognizing IMPS as a spectrum, the guidelines reflect the clinical reality that myocarditis and pericarditis can coexist. This approach acknowledges their shared etiologies, infectious, autoimmune, and autoinflammatory, and their anatomical and clinical overlap. Framing IMPS as a continuum rather than isolated entities underscores the need for inclusive evaluation perspectives. This translates directly into the everyday routine of CMR imagers: a comprehensive review and description of the myopericardial CMR pathology is crucial for the clinical utility of the CMR examination.

### 2.2 Patient-oriented, presentation-driven pathways

A major innovation in the 2025 ESC Guidelines is the introduction of symptom-driven diagnostic and therapeutic algorithms. Instead of rigid disease categories, the guidelines propose pathways based on clinical presentation, considering chest pain, shortness of breath, or arrhythmic events. This presentation-centered approach streamlines the assessment of disease severity and differential diagnosis in the individual physiological and clinical context, informing clinical decision-making, particularly in acute settings, including cardiac tamponade or constrictive physiology. In nearly all presented flowcharts, CMR plays a central role, from diagnosis to risk stratification, and finally guiding treatment and further workup. Of specific importance is the unique ability of CMR to discriminate myocardial inflammation from other etiologies of acute myocardial injury and to identify likely causes thereof, such as secondary cardiomyopathies like sarcoidosis, rheumatologic disorders with cardiac involvement, Takotsubo cardiomyopathy, myocardial injury without coronary artery stenosis, and others. Virtually all patients with IMPS need a CMR within their patient journey, including follow-up scans. In selected patients with strong clinical evidence for predominant pericarditis, clinical assessment and blood markers may, however, be sufficient.

### 2.3 Genetics, immunity, and inflammation

The guidelines also emphasize the interplay between genetics, autoinflammation, and autoimmunity. For patients with recurrent pericarditis or myocarditis and associated risk features (family history of IMPS, cardiomyopathy, inflammatory phenotype, complicated course, clinical and imaging findings raising suspicion of an inherited heart disease), genetic testing is suggested to identify overlapping cardiomyopathic or autoinflammatory traits. These insights may reshape our understanding of IMPS, moving it from isolated, single etiologies (e.g., infectious, toxic) to overlapping conditions that may include immune-mediated and genetic heart diseases. Special care is needed in patients with recurrent forms of IMPS, where a raised suspicion for overlapping conditions is warranted. Comparing the CMR results with previous scans provides important details regarding the temporal change of findings, such as previous inflammation or ventricular functional adaptations, and should be part of good clinical practice.

### 2.4 Red flags for early recognition

Timely identification of high-risk patients with IMPS remains critical. The guidelines delineate red flags, including symptoms, signs, biomarkers, or imaging findings suggesting an IMPS, to support earlier diagnosis and treatment. By raising awareness of these warning signs, the guidelines aim to reduce diagnostic delays and improve patient outcomes. It is well-known that running a CMR exam as early as possible after presentation of suspected IMPS can increase the diagnostic capability.

### 2.5 The paradigm shift toward non-invasive imaging

**Fig. 1:**
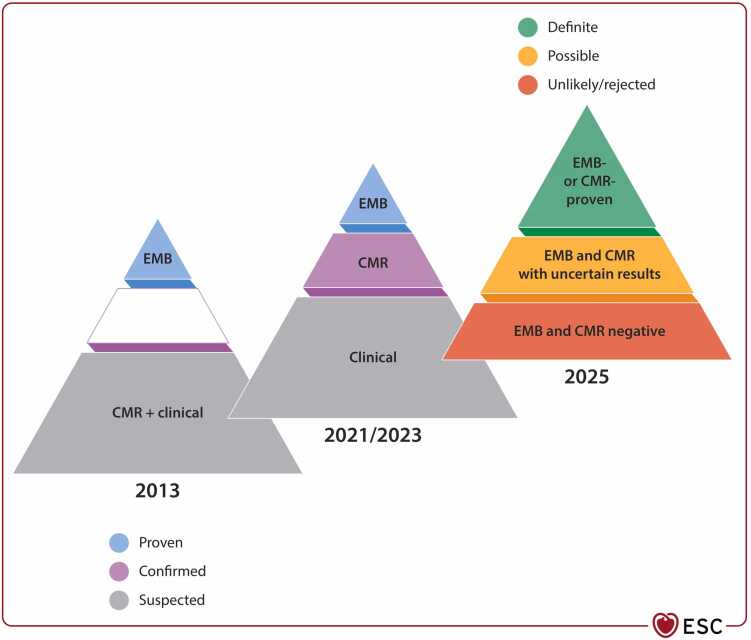
The paradigm change in the clinical diagnosis of myocarditis (License number: 6124750295099). *CMR* cardiovascular magnetic resonance, *EMB* endomyocardial biopsy

Perhaps the most profound change in the 2025 ESC guidelines, compared to the previous 2013 ESC consensus statement on myocarditis, relates to the role cardiac imaging plays in IMPS [1], [3]. CMR now stands at the center of IMPS evaluation, enabling definite non-invasive diagnosis in uncomplicated myocarditis and providing detailed phenotyping through advanced tissue characterization, including parametric mapping and late gadolinium enhancement. While clinical data, electrocardiogram (ECG), blood biomarkers, and echocardiography still play important roles in the routine management of these patients, CMR allows for a comprehensive assessment that is now on par with endomyocardial biopsy (EMB) in terms of clinical utility in IMPS. This represents a true paradigm shift: in many patients, CMR obviates the need for invasive diagnostics while still enabling precise therapeutic guidance (Fig. 1). It is important to mention that a “CMR-proven” diagnosis can be achieved only when using the updated Lake Louise Criteria, as the body of published scientific literature indicates that the diagnostic accuracy is higher in comparison to the classical approach [4], [5].

### 2.6 A revised role for endomyocardial biopsy

While CMR has expanded its diagnostic power, EMB remains important in selected cases of myocarditis, especially in hemodynamic unstable patients. The guidelines clearly define the use of EMB in intermediate-to-high-risk patients in whom histopathological data may guide therapy, such as fulminant myocarditis, myocarditis with arrhythmic or heart failure presentations, or suspected specific etiologies, such as giant cell myocarditis. This more focused use of EMB reflects a balance between non-invasive precision and the irreplaceable value of a confirmed histotype in selected cases.

### 2.7 Individualized exercise prescription

The traditional “one-size-fits-all” approach to exercise restriction has been replaced by individualized recommendations. The decision to resume physical activity, whether sports or work, is now guided by disease phenotype, severity, and follow-up imaging. CMR plays a central role in monitoring recovery, ensuring safe reintegration, and preventing adverse outcomes in vulnerable patients.

### 2.8 Therapeutic strategies in IMPS

The management of myocarditis follows a tiered approach: uncomplicated cases are treated with anti-inflammatory therapies, while more complex scenarios require etiology-specific treatments. For high-risk patients, advanced interventions may include mechanical circulatory support or temporary devices, such as wearable cardioverter-defibrillators. Again, cardiac imaging, and CMR in particular, guides clinical management, risk stratification, monitoring of the response to treatment, and decision-making in specific clinical scenarios such as indications for device therapy. The therapeutic algorithm for pericarditis has been substantially updated. In addition to conventional anti-inflammatory therapies, new options include anti-interleukin-1 agents for recurrent or refractory cases. Such novel therapies mark a significant step forward in tailoring treatment to disease biology and patient need. In certain cases of pericarditis, CMR can be of value in defining a remission, as low levels of blood inflammatory biomarkers can be false-negative.

### 2.9 Multidisciplinary approach as standard of care

Finally, the guidelines underscore the need for multidisciplinary collaboration in the management of more severe and complicated cases of IMPS. Cardiologists, electrophysiologists, rheumatologists, infectious disease experts, pathologists, and imaging specialists all play essential roles. CMR imagers are integral to the team, as they have become a cornerstone for diagnosis and follow-up of IMPS. Shared decision-making, patient education, and transparent communication are highlighted as key to improving patient adherence to treatment and long-term outcomes. Early referral to hub centers is suggested for complicated cases that cannot be managed in spoke centers.

## 3 The special role of CMR and call for quality

**Fig. 2:**
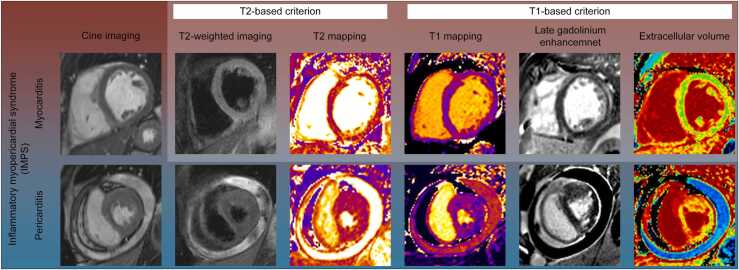
The spectrum of the inflammatory myopericardial syndrome and the updated Lake Louise Criteria

The CMR community has always been characterized by a strong spirit of experimentation and innovation. This creativity is essential for driving progress, advancing technology, uncovering new pathophysiological mechanisms, and ultimately guiding therapy. At the same time, we are also called upon to establish standards and deliver reliable, reproducible results. This includes transparent reporting of limited diagnostic confidence, maintaining a constant awareness of potential sources of error. Standardization and quality assurance of CMR methods should be achieved in collaboration with industry partners, with a common goal to deliver the best care to patients. In the context of IMPS, the comprehensive nature of CMR has made the technique central in algorithms. The guidelines also highlight, in line with the updated Lake Louise Criteria (Fig. 2), the importance of including T2-based techniques for the assessment of inflammatory activity. Consensus papers from the Society for Cardiovascular Magnetic Resonance provide an important framework [6], [7]. If mapping is not available, or if the data are limited or non-diagnostic, the findings should be classified as “possible” or “uncertain,” rather than definitive. Importantly, CMR should always be interpreted as part of a broader clinical assessment. The value of CMR lies in the multiparametric approach, providing numerous biomarkers with one scan. While some are well-established, for example, functional and volumetric assessment, and necrosis/fibrosis on late gadolinium enhancement, other emerging imaging biomarkers will change and enrich our understanding of disease pathways, such as mapping and deformation assessment. The global and holistic interpretation of these findings will enable CMR to be the imaging method of choice in the assessment of these patients.

For CMR to be the imaging modality of choice in IMPS, as is now recommended in the guidelines (Table 1), it is necessary to consistently provide high-quality, reliable, and relevant data. While the technology is robust, this calls for access to CMR-ready scanners, trained technologists, and experienced readers. The ESC guidelines on IMPS are a decisive step toward a non-invasive imaging-guided diagnostic pathway, which is anticipated to feature in more upcoming guidelines to empower this approach, for example, such as in the 2024 ACC Expert Consensus Decision Pathway on Strategies and Criteria for the Diagnosis and Management of Myocarditis [8]. Further prospective outcome studies will likely move the Level of Evidence for CMR in IMPS to A, but studies on the impact of using CMR on outcome must be sufficiently large and properly designed. This should be a joint effort and priority of the CMR and medical community.

**Table 1:** Recommendation of the 2025 ESC guidelines for the management of myocarditis and pericarditis, including CMR (Adopted from [1]).

Recommendation	Class	Level
CMR is recommended in patients with the clinical suspicion of myocarditis (using updated LL criteria) and/or pericarditis for the non-invasive diagnosis of inflammatory reaction.	I	B
CMR is recommended in patients with suspected myocarditis to reach a clinical diagnosis and to determine the cause of acute myocardial injury, including assessment of edema, ischemia, and necrosis/fibrosis/scarring.	I	B
CMR is recommended for follow-up at least within the first 6 months in patients with myocarditis to identify a healed or ongoing process, for risk stratification and personalized therapy, and to enable a return to exercise.	I	C
CMR is recommended in patients with suspected pericarditis when a diagnosis cannot be made using clinical criteria to assess evidence of pericardial thickening, edema, LGE, and to assess the persistence of disease during follow-up in selected cases.	I	B
Anti-IL-1 agents (anakinra or rilonacept) should be considered in cases of incessant/recurrent pericarditis with evidence of pericardial inflammation on CMR after failure, contraindications, and intolerance to first-line therapies and corticosteroids, regardless of C-reactive protein levels to reduce recurrences and allow corticosteroid withdrawal.	IIa	C
ICD implantation should be considered in patients with non-active^a^ myocarditis and hemodynamically tolerated sustained VT to prevent SCD.	IIa	C
ICD implantation may be considered in patients with myocarditis after the acute phase (3–6 months) and persistent risk factors for VA^b^ to prevent SCD	IIb	C
Follow-up with clinical assessment, biomarkers, ECG, exercise test, Holter-ECG monitoring, echocardiography, and CMR at least within 6 months after the index hospitalization is recommended in all patients with myocarditis to identify a potential progression or new risk factors.	I	C
CMR, using tissue characterization techniques, is recommended in patients with suspected CS to assess cardiac inflammation and myocardial involvement.	I	C
ICD implantation should be considered in patients with CS and LVEF >35% after resolution of the active phase with significant LGE, a history of arrhythmias, unexplained syncope, inducible sustained VA at PVS, or with persistent high-degree AVB to prevent SCD.	IIa	C

*CMR* cardiovascular magnetic resonance, *ECG* electrocardiogram, *LL* Lake Louise, *LGE* late gadolinium enhancement, *IL-1* interleukin-1, *ICD* implantable cardioverter defibrillator, *VT* ventricular tachycardia, *SCD* sudden cardiac death, *VA* ventricular arrhythmias, *CS* cardiac sarcoidosis, *LVEF* left ventricular ejection fraction, *PVS* programmed ventricular stimulation, *AVB* atrio-ventricular block, *NSVT* non-sustained ventricular tachycardia

a Non-active based on CMR evidence of activity (T2)

b NSVT, extensive LGE, unexplained syncope, positive PVS, reduced LVEF <50%

## 4 Conclusion

The 2025 ESC guidelines on IMPS represent a landmark achievement in our current approach to inflammatory heart disease. They provide an umbrella concept that bridges myocardial and pericardial inflammation, introduce presentation-driven management algorithms, and redefine the roles of EMB and genetics in the diagnostic workup. At the heart of this new framework is multimodality imaging, with CMR as the central tool for the non-invasive verification, risk stratification, and follow-up care of patients with IMPS. For the CMR community, this not only validates the progress of the past decades but also challenges us to further refine techniques and standardize CMR methodologies in collaboration with industry partners, prescribe consistent and efficient imaging protocols, provide high-quality reports, and expand access. The 2025 ESC guidelines’ message is clear: CMR is no longer supportive—it is decisive.

## Declaration of competing interests

The authors declare that they have no known competing financial interests or personal relationships that could have appeared to influence the work reported in this paper.
